# How to make a porphyrin flip: dynamics of asymmetric porphyrin oligomers†

**DOI:** 10.1039/c5cp04636j

**Published:** 2015-09-15

**Authors:** Cheng Shang, Julian M. Philpott, Nick Bampos, Paul D. Barker, David J. Wales

**Affiliations:** a University Chemical Laboratories Lensfield Road Cambridge CB2 1EW UK cs778@cam.ac.uk dw34@cam.ac.uk

## Abstract

We present the first predictions of *meso*-aryl flipping pathways in porphyrin oligomers. In the context of cyclic oligoporphyrins this flipping results in a paddle rotation of each porphyrin monomer in the oligomeric ring. If the monomer porphyrin units are asymmetric, this flipping will have consequences for their supramolecular behaviour. Desymmetrisation of synthetic porphyrins leads to synthetic challenges, and hence these species are not as well studied as the more accessible, symmetric counterparts. We have both simulated and synthesized novel, desymmetrised monomeric and cyclic trimeric porphyrins and we predict that the flipping barrier for a porphyrin monomer within the trimer is 36.7 kJ mol^−1^ higher than that for *meso*-aryl flipping in the monomer. The flipping rates estimated from Variable temperature NMR data are consistent with these results. We have also carried out a systematic investigation of how porphyrinic substituents will affect the dynamics, revealing that adding steric bulk in the right place can facilitate *meso*-aryl flipping. While supramolecular chemistry often focuses on highly symmetric assemblies, evolution can break molecular symmetry in subtle ways, leading to many pseudosymmetric assemblies in biology, especially protein–porphyrinic complexes that are important for energy harvesting and electron transport systems. The dynamic behaviour we have characterized can be critical for the design and function of these molecules, and hence our results will help inform future efforts in the synthesis of asymmetric porphyrinic assemblies that interact with biomolecules.

## Abstract 2

The pathways and energy barriers from computational and experimental methods reveal the dynamics of asymmetric porphyrin oligomers.

## Graphical abstract

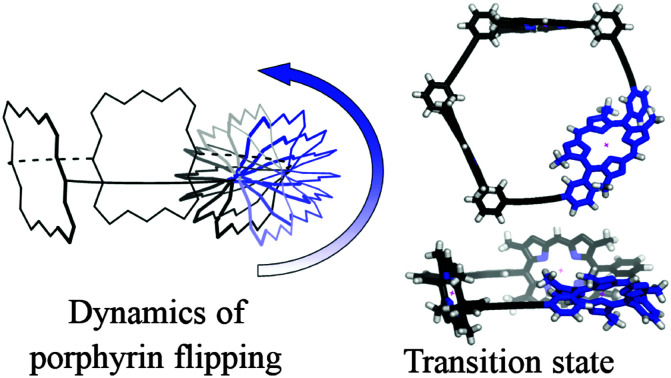

## 1 Introduction

Consideration of dynamic behaviour is critical in the design and function of supramolecular assemblies, allowing complex architectures to form,^1–3^ which may then mediate selective binding and catalysis.^4–7^ Organised arrays of porphyrins play important roles in nature, especially in photosystems I and II, and light harvesting systems 1 and 2.^8–10^ Circular porphyrin arrays also play important roles in recognition, light harvesting, and catalysis for supramolecular chemistry.^5,11,12^ In natural systems porphyrinic cofactors are organised by the protein structure through metal ligation and hydrophobic interactions, with no direct covalent linkage between porphyrins, in contrast to many artificial porphyrinic arrays.^13–18^ Does nature avoid linkage between these abundant prosthetic groups, or has it not discovered such structures yet? If naturally evolved porphyrins are yet to sample covalently linked structures, then it will be interesting to ask how we might integrate and exploit such non-natural prosthetic groups within natural systems and what structural and dynamic factors are important. In this work we present a desymmetrised analogue of a well-studied cyclic porphyrin oligomer, which was designed to aid our understanding of porphyrin dynamics. Computational analysis is employed to provide insights that have been proven challenging to obtain from experimental measurements alone.

Many studies of porphyrin dynamics have focused on distortions of the core conformation, *e.g.* the characterization, the origin and their impact on the ligand binding to the central metal.^19–22^ Some other work has addressed synthesis of a series of oligomers with various cavity sizes and flexibilities by varying the linker between porphyrins.^23,24^ The rotation of *meso*-aryl in the porphyrin monomer has been probed using both experiments and simulations for different substituents at the *axial*-, *meso*- and β-pyrrolic positions.^25–30^ Substituent effects at these sites are likely to be significant because of their impact on the geometric and electronic structures for putative supramolecular devices. In particular, the *meso*-aryl rotation is very important in the synthesis of unsymmetrical porphyrins and the corresponding oligomers. In 1975, Eaton *et al.* synthesized a series of tetraphenylporphyrins with different central metals and alternative substituents at the *para*-position of *meso*-aryl.^25^ The rotational barrier of the *meso*-phenyl group that was reported from variable temperature NMR was around 62 to 75 kJ mol^−1^. Similar results have also been obtained in the last decade using NMR, molecular mechanics, and higher level calculations.^26–29^ However, to the best of our knowledge, there have been no investigations into the flipping of a single macrocycle in a porphyrin oligomer.

**Fig. 1:**
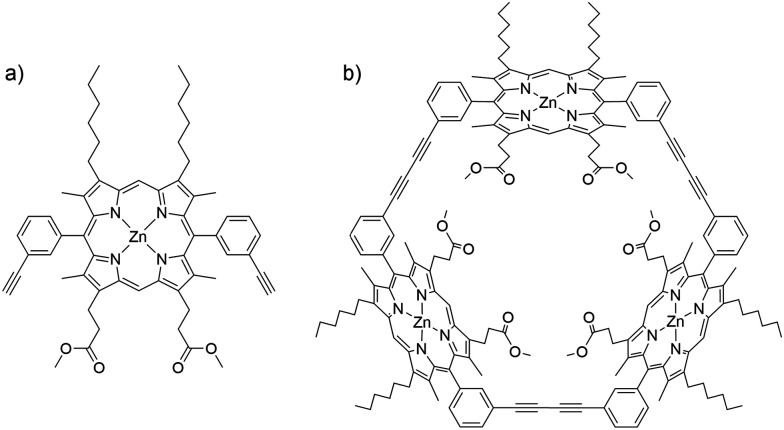
Structures of (a) ZnBAP_*m*_ (P1) and (b) Zn_3_TRI_*m*_ (P2).

In the present contribution, we combine experiment with analysis of pathways and rates, including explicit treatment of electronic structures, exploring the flipping of a porphyrin monomer, dimethyl 3,3′-(5,15-bis-(3-ethylphenyl)-8,12-dihexyl-3,7,13,17-tetramethyl zinc porphyrin-2,18-diyl) dipropionate (ZnBAP_*m*_, P1) in its cyclic trimer, Zn_3_TRI_*m*_ (P2, Fig. 1). We find that the flipping barrier of the monomer macrocycle in P2 is 36.7 kJ mol^−1^ larger than that associated with *meso*-aryl rotation in P1. A systematic investigation of the steric effect at the *meso*- and β-pyrrolic positions on the *meso*-aryl rotation in the monomer was also performed. A large substituent at the β-pyrrolic position is shown to decrease the flipping barrier, with important implications for the design and synthesis of porphyrin oligomers.

## 2 Computational details

The basin-hopping global optimisation algorithm^31–33^ implemented in our GMIN program^34^ was used to predict the initial structures of P1 and P2. The doubly^35,36^ nudged elastic band (DNEB) method,^37^ as implemented in the OPTIM program,^38^ was used to generate likely candidate structures for transition states (TSs) along pathways of interest. These candidate structures were then refined accurately using hybrid eigenvector-following.^39,40^ The two minima that each TS connects were identified by calculating approximate steepest-descent paths.

Both the AMBER ff99SB^41–43^ molecular mechanics force field and quantum mechanical (QM) calculations with explicit treatment of electronic structures were used to analyse the potential energy surface. The partial charges used to generate the AMBER force field were calculated using GAMESS^44^ at the B3LYP/6-31G* level^45–48^ by the restricted electrostatic potential method.^49^ The force field parameters are a combination from the general AMBER force field (gaff) parameter set,^50^ parameters for heme from the AMBER parameter database,^51^ and parameters generated by Lin and Wang for zinc-containing compounds.^52^ Initially, potential energy surface exploration was performed for the AMBER potential. The resulting minima and transition states were refined using the Gaussian 03 software package^53^ interfaced to OPTIM. The P1 and P2 optimization used the B3LYP functional and 6-31G(d,p) basis sets first, followed by single point energy calculations using 6-31++G(d,p) to improve the possible nonbonded interactions. The 6-31G(d,p) basis set has been used to investigate *meso*-aryl rotation in small porphyrin monomers.^26^ Then, another single point energy calculation with chloroform was represented by the polarizable continuum solvent model (PCM)^54,55^ in Gaussian 03. In the subsequent analysis, optimization of all the monomers was performed at the B3LYP/6-31++G(d,p) level of theory in vacuum. The zero point energy was calculated using the AMBER force field for both P1 and P2.

## 3 Results and discussion

We present a detailed analysis of the predicted pathways between conformational isomers for a number of porphyrin systems. Our investigation begins with the novel porphyrin P1 and is extended to the cyclic trimer, P2, whose synthesis and NMR characterisation have allowed us to experimentally corroborate the computational results. In addition, we present a systematic study of β-pyrrolic and *ortho*-aryl substitution on the energy barriers for conformational exchange to inform future synthetic efforts.

### 3.1 *meso*-Aryl flipping mechanism in P1

#### 3.1.1 Predicted characteristics of the flipping pathway

**Fig. 2:**
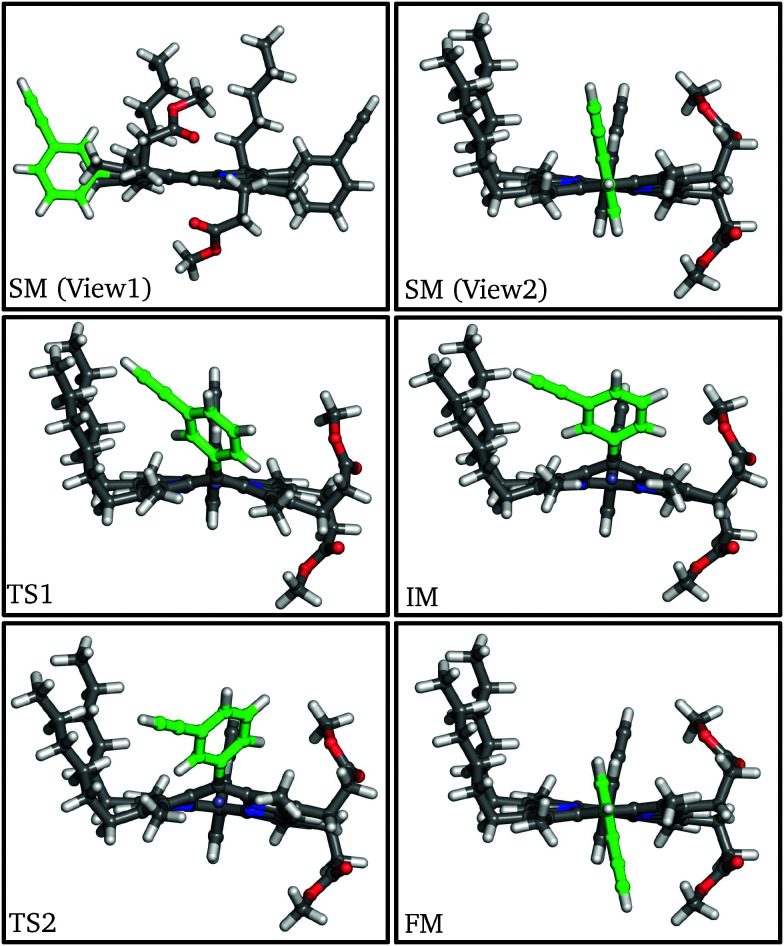
Structures of stationary points along the *meso*-aryl rotational pathway for P1, highlighting the rotating aryl ring (green).

The global minimum of P1 employed as the starting minimum (SM) is the *syn*-conformer with respect to the two 3-ethynyl *meso*-aryl groups. Starting from this conformation the rotational pathway about the *meso*-aryl bond was calculated. The structures of key stationary points were identified (Fig. 2) and the corresponding energies are tabulated (Table 1). We note that in the initial structure the porphyrin macrocycle is slightly buckled, corresponding to a ruffle distortion.^56^ The β-hexyl groups are almost parallel to each other, with the β-ester groups antiparallel. The aryl substituents align perpendicular to the plane of the *meso*- and α-pyrrolic carbons.

**Table 1:** Potential energy of each stationary point along the path corresponding to the starting minimum (kJ mol^−1^)

	Δ*E*^a^	Δdiff^b^	Δsolv^c^	ΔZPE^d^	Final^e^
SM	0.0	0.0	0.0	0.0	0.0
TS1	64.4	+1.9	−1.3	−1.9	63.1
IM	59.0	+3.3	−1.1	−3.0	58.2
TS2	62.0	+1.7	−0.7	−2.3	60.7
FM	0.4	+0.5	−0.6	0.0	0.3

a Potential energies evaluated using a 6-31G(d,p) basis set.

b Energy differences contributed by the diffuse basis set.

c Energy differences contributed by the solvent effect.

d Energy differences contributed by the zero point energy.

e The sum of all the terms.

**Fig. 3:**
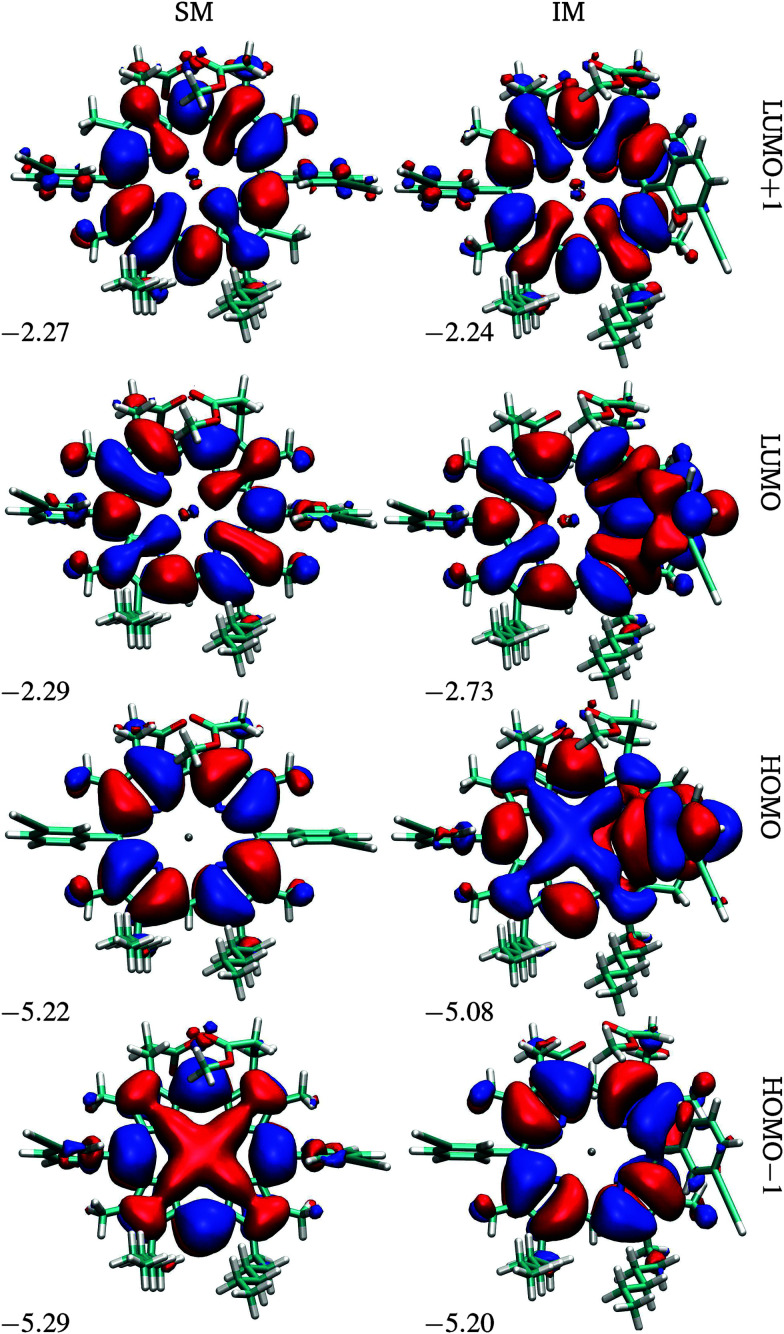
Frontier orbitals of P1 in the starting and intermediate minima, SM and IM. The energies are in eV.

At the first transition state (TS1), significant distortion of the porphyrin ring is observed, which lifts the aryl group to a dihedral angle of 41.5°, with respect to the plane of the macrocycle. This distortion coincides with a small offset in the β-methyl substituents in the opposite direction. Thus, the steric clash between the *ortho*-protons and the β-methyl moieties is relieved in this transition state. The intermediate minimum (IM) exhibits a similar configuration and only a slight energy reduction relative to TS1 (Table 1). In the intermediate minimum the aryl group becomes coplanar with the porphyrin ring, producing a striking difference in the electronic character: conjugation extends over the aryl ring, inverting the energies of the HOMO and HOMO−1, as well as breaking the degeneracy of the LUMO and LUMO+1 (Fig. 3).

The second half of the pathway is similar to the first half in both energy and structure, resulting in a final minimum (FM) where the two aryl groups are now *anti* to each other. Other than this *anti* configuration, the final minimum is almost identical to the initial minimum, and the energy profile of the whole process is roughly symmetrical and thermoneutral, with a barrier of 63.1 kJ mol^−1^, which corresponds to a rate constant of 63.8 s^−1^ at 300 K according to transition state theory for harmonic normal mode frequencies. The energy barrier and the conformational changes presented here are consistent with our experimental results from variable-temperature NMR (VT-NMR) spectroscopy (see below), as well as previous experiments and simulations.^25–29^

#### 3.1.2 VT-NMR spectroscopy of P1 and P3

**Fig. 4:**
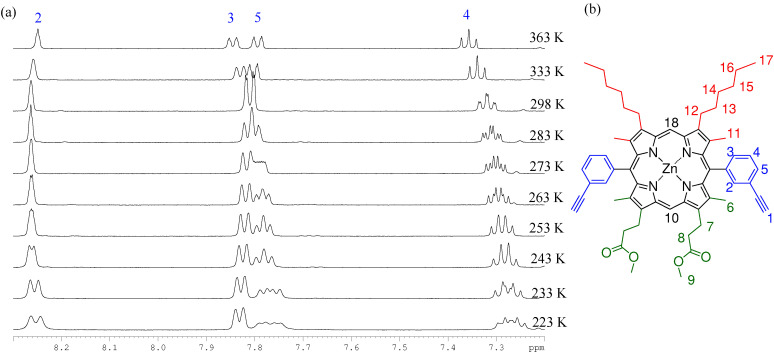
(a) ^1^H VT-NMR spectra collected in the aromatic region of P1 (d_8_-toluene, temperatures ranging from 223–363 K). (b) The structure and assignment of P1.

Proton NMR of P1 (298 K in d_8_-toluene) produces a well-defined and predictable spectrum, with one subtle discrepancy: the resonance at 7.35 ppm appears as a doublet of triplets where a triplet would be expected for this proton environment (peak 4 in Fig. 4). Upon increasing the temperature the expected triplet is observed. However, when the temperature is lowered a significant change occurred, as the triplet deconvolves into two signals. At 223 K seven aryl resonances are observed, in place of the expected four, using COSY and TOCSY analysis (ESI†) suggesting the presence of two different spin systems. Only the resonance associated with proton 5, which is coaxial with the *meso*-aryl bond, does not deconvolve into two signals and remains common to both spin systems.

We interpret this behaviour in terms of interconversion between the *syn* and *anti* atropisomers that arise due to hindered rotation around the *meso*-aryl bond. Typical first order coalescence behaviour is not seen in this system, which may be due to a dependence of the rate of *meso*-aryl rotation on the rate of porphyrin ring buckling, giving rise to the observed changes in shift rather than classical coalescence, similar to the work published by Deeming *et al.*^57^

To confirm that this observation is not a result of the asymmetrical nature of P1 these experiments were also conducted on ZnBAP_*b*_ (P3), the symmetrical tetra-hexyl analogue of P1, which was prepared according to the literature method.^58^ Very similar temperature dependent behaviour was observed, demonstrating that the β_2_ substitution has little effect. Spectra collected for P1 in d_5_-pyridine also produce the same results, ruling out facial stacking and porphyrin aggregation phenomena as the origin of the deconvolution of aryl signals. We have considered whether these observations might be a result of thermal averaging that prevents the resolution of the locked atropisomers at increased temperatures, as opposed to our hypothesis that the NMR timescale is around that of the rotation rate. However, reported yields (50 to 55%)^59^ for the synthesis of a cyclic trimer are inconsistent with a system containing conformationally locked atropisomers, where only the *syn* form is disposed to form the cyclic trimer.

Δ*G*^‡^ for rotation was estimated as Δ*G*^‡^ = *aT*[9.972 + ln(*T*_C_/Δ*u*)], where Δ*G*^‡^ is the Gibbs free energy required for rotation at temperature *T*, *a* is a constant equal to 1.914 × 10^−2^ kJ mol^−1^, *T*_C_ is the temperature of coalescence, and Δ*u* is the frequency difference of the chemically equivalent resonances in the slow exchange limit.^60^*T*_C_ was obtained by plotting Δ*u versus T* for the dynamic range in the VT-NMR and the results for Δ*G*^‡^ are presented in Table 2. From these data we estimate Δ*G*^‡^ for rotation about the *meso*-aryl bond to be 65.4 kJ mol^−1^. This value is in reasonable agreement with the calculated energy barrier of 63.1 kJ mol^−1^.

**Table 2:** Experimentally derived Gibbs free energy of *meso*-aryl rotation for P1

Proton assignment	Δ*G*^‡^ (298 K)/(kJ mol^−1^)	Error
1	66.8	0.65
2	65.1	1.81
3	64.9	19.07
4	64.8	1.20
Average	65.4	

### 3.2 Rotational pathway in the cyclic porphyrin trimer, P2

#### 3.2.1 Predicted flipping pathway

**Fig. 5:**
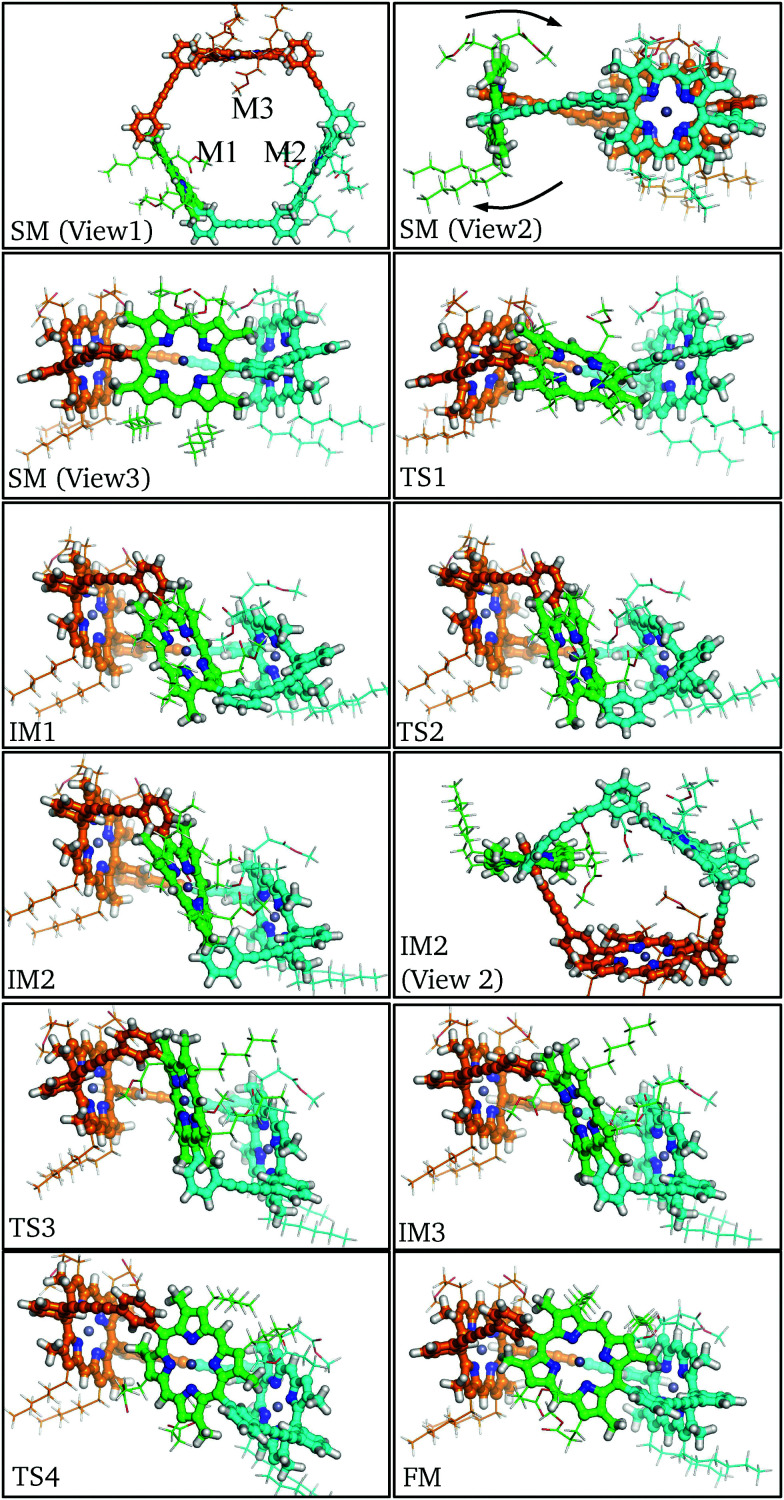
Key structures along the rotational pathway for P2. The monomeric units are identified as the rotating M1 (green), static M2 (cyan), and static M3 (orange). For clarity, the aryl substituents of M1 are coloured as for the adjacent M2 and M3. The flipping direction is labelled in the starting minimum (view2).

An initial structural model for P2 was obtained by the removal of alkynyl protons and the combination of three P1 monomers (Fig. 5 and videos, see ESI†). The relaxed structure closely resembles the crystal structure of a previously reported related cyclic trimer.^61^ In this *C*_3_ symmetric oligomer the three units are identical and preserve the monomeric structure closely. Subsequent global optimization using GMIN and the AMBER force field identified several new local minima, which were proved to be less stable than the initial structure when relaxed using explicit treatment of the electronic structure. Hence the original structure was used as the starting minimum (SM) for the pathway analysis. For convenience the rotating monomer is labelled M1 and the other monomers, M2 and M3, passively adjust their configurations to make room for the M1 flipping (Fig. 5). For the initial minimum the individual porphyrin macrocycles are perpendicular to the trimer equator, so M1 could follow two inversion paths. Here we focus on the rotation that makes the β-pyrrolic hexyl chains remain on the exterior, whilst the β-pyrrolic ester groups traverse the trimer interior. The other path is very similar to this one but with a slightly higher energy barrier, probably due to the differences in steric interactions.

In order for the rotation of M1 to occur both aryl rings must rotate about the *meso*-aryl bond, changing from the *syn*-conformation *via* the *anti*-conformation (IM2) back to the *syn*-conformation. The local configuration of M1 at TS1, IM1 and TS2 closely matches the corresponding stationary points in P1: C_β_–C_*m*_–C_β_ distorts out of the porphyrin plane and the β-methyl groups bend in the opposing direction to allow aryl ring rotation. No significant orientational or conformational changes in M2 and M3 are observed until IM1, at which point the aryl rings of M1 are perpendicular, causing M2 and M3 to adjust their positioning, while the butadiene linkers bend to accommodate the strained configuration. This bending manifests itself as a tilt in the rotational axis of M1 by around 45° from the starting minimum to intermediate minimum IM1.

Following the pathway from IM1 to IM3 reveals little change in the tilt of the M1 rotational axis, and the M2 and M3 conformations remain relatively constant. This section of the pathway makes the flip and inversion of M1 *via* IM2, which is analogous to the final minimum for the P1 pathway. Two notable differences are observed: (i) the *anti*-aryl groups are now closer to perpendicular, creating a torsional angle of about 45° along the *meso*-aryl bond, which serves to accommodate the conformation restraints imposed by M2 and M3. (ii) M1 is more buckled than the monomer in the *anti*-conformer, in contrast to the closer planarity of both M2 and M3. The greater strain at intermediate minimum IM2 results in destabilization relative to the starting minimum by 37.8 kJ mol^−1^.

Beyond intermediate minimum IM2 a nearly identical reverse pathway is observed, but with rotation about the second aryl ring. In the final minimum the *C*_3_ symmetry is broken relative to the starting minimum, due to the exchange of the β-pyrrolic hexyl and methyl-propionate side chains, resulting in *C*_s_ symmetry. There are other subtle configurational differences, especially the increased buckling of M1 and decreased buckling of M2. The result of these changes is that the final minimum is 6.8 kJ mol^−1^ higher in energy than the initial minimum. The highest energy barrier found corresponds to TS2 at 101.5 kJ mol^−1^, with an increase of 37.6 kJ mol^−1^ relative to the monomeric case. This significant energy barrier increase is predicted to reduce the rate constant for rotation in the trimer to around 10^−5^ s^−1^ at 300 K.

The intrinsic barrier difference between P1 and P2, *i.e.* Δ*E* for TS1 in Tables 1 and 3, which is caused by the conformational constraint between monomers in P2, is only 16.2 kJ mol^−1^. The diffuse basis set and the solvent effect contribute little to the flipping barrier in P1, but change the energy of IM1 and TS2 significantly. The energy increase caused by the diffuse basis set (Δdiff) is due to the repulsion between two β-pyrrolic ester groups of M1 and M2 during M1 flipping. The largest Δdiff corresponds to IM1 and TS2 in P2 (+13.3 kJ mol^−1^) and decreases with the repulsion between ester groups. The energy increase caused by the solvent effect (Δsolv) is a result of the burial of the β-pyrrolic ester groups within the macrocycle, thus reducing the surface area and the polarity of the molecule compared to the initial minimum. Notably, the same striking conjugation of the *meso*-aryl orbitals with the porphyrin core seen in IM of P1 (Fig. 3) is observed in the HOMO of IM1 and IM3 in similar calculations of P2 (see ESI†).

**Table 3:** Potential energy of each stationary point along the predicted pathway for rotation in P2 (kJ mol^−1^)

	Δ*E*	Δdiff	Δsolv	ΔZPE	Final
SM	0.0	0.0	0.0	0.0	0.0
TS1	80.6	+4.7	+6.0	−3.1	88.2
IM1	64.2	+13.3	+9.9	−1.3	86.1
TS2	76.3	+13.3	+10.5	+1.2	101.5
IM2	18.3	+10.9	+6.8	+1.7	37.8
TS3	81.3	+7.5	+5.2	+0.4	94.4
IM3	67.0	+7.2	+4.3	−1.2	77.2
TS4	82.5	+4.0	+2.9	−0.9	88.5
FM	2.6	+3.0	+1.2	0.0	6.8

#### 3.2.2 VT-NMR spectrum of P2

The design of P2 was intended to provide insight into the rotational behaviour of monomeric units within oligomeric macrocycles. The peripheral protons in the system were expected to act as markers to report on the conformational state of the oligomer, which has two distinct orientations in the low energy state, P2-SM, where porphyrin units present their β-pyrrolic side chains unidirectionally, or P2-FM, where one unit directs its β-pyrrolic side chains antiparallel to the other two units. For example, in the initial minimum we expected two *meso* proton signals, and in the final minimum four, a total of six *meso* environments for a statistical mixture of the P2 local minima corresponding to slow exchange.

The ligand 2,4,6-tri(pyridine-4-yl)-1,3,5-triazine, TPyT, was added to obtain a spectrum of P2 in a rotationally locked state. TPyT is known to bind with chelate cooperatively into analogous cyclic zinc porphyrin trimers with association constants between 9 × 10^9^ and 4 × 10^10^ mol^−1^ dm^3^ through nitrogen-zinc ligation.^62^ In this TPyT bound configuration, it is assumed that the porphyrin units are fixed and prevent monomer inversion.

**Fig. 6:**
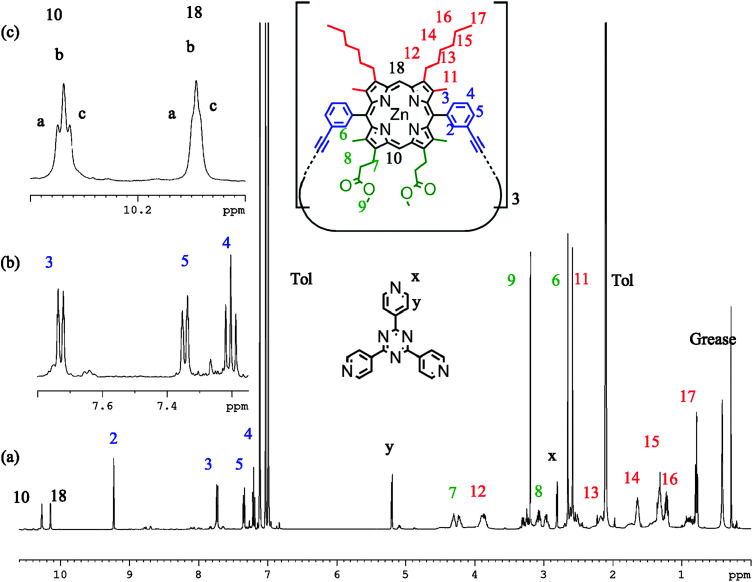
^1^H NMR spectra of P2 with TPyT bound (298 K, d_8_-toluene, 700 MHz) showing (a) the proton assignment, (b) expanded regions of the aryl signals, and (c) the *meso* environments.

^1^H NMR spectra of P2 (d_8_-toluene, 298 K) with TPyT bound exhibit six *meso* proton environments in addition to a deconvolution of signals attributed to the alkyl and ester side chains (Fig. 6). Signals at 2.80 and 5.20 ppm are characteristic of heavily shifted (lower ppm) TPyT signals caused by binding within the trimer; there is no observable unbound TPyT in solution. Therefore it is clear that porphyrin unit flipping is not seen on this time scale and we observe a statistical mixture of P2-IM and P2-FM. If the temperature is increased, resolution of the six *meso*-environments becomes difficult, the TPyT signals broaden, and separate side chain signals are no longer distinguishable. However, there remains no evidence that TPyT becomes unbound from P2, suggesting that thermal averaging hampers the distinction between P2-IM and P2-FM, but does not facilitate interconversion (ESI†). Cooling this sample (min. 223 K) produces no significant differences in comparison to the measurement at 298 K. At all temperatures not more than four aryl signals were characterised, suggesting that only the *meso*-aryl *syn* conformation is observable, in view of the other evidence presented above.

The same sample in d_5_-pyridine shows displacement of TPyT by the solvent, two apparent *meso* signals, no deconvolution of side chain resonances, and four aryl signals. Variable temperature NMR of this system shows no deconvolution or sharpening of proton resonances. We ascribe this behaviour to a system where P2-IM and P2-FM are both present in solution, but are not interconverting. However, they remain indistinguishable by NMR due to thermal averaging of the peripheral protons, which can sample many more environments without the locking influence of the TPyT ligand. If this is the case, the energy barrier for flipping is significantly higher for P2 than the energy barrier of rotation is for P1.

### 3.3 The effect of substituents on *meso*-aryl flipping

For a more general, systematic study we have considered the effect of a range of β-pyrrolic substituents on aryl group rotational barriers. The aim here is to guide the modification of the porphyrin to facilitate or restrain aryl motion, which is important for synthesis, to prevent scrambling of moieties, and for twisting oligoporphyrin properties. The results are organised according to modification at the two β-pyrrolic positions and the *ortho*-position on *meso*-aryl (Table 4, Fig. 8).

**Table 4:** The energy barrier for *meso*-aryl flipping with different substituents at the β_1_, β_2_ and *ortho* positions corresponding to P1 (kJ mol^−1^)

	β_1_	β_2_	*ortho*	Barrier
1	H	H	H	77.1
2	CH_3_	H	H	75.6
3	C_2_H_5_	H	H	70.1
4	NO_2_	H	H	53.8
5	CF_3_	H	H	48.8
6	CCl_3_	H	H	27.8
7	C(CH_3_)_3_	H	H	30.4
8	H	C_2_H_5_	CH_3_	182.7
9	CH_3_	C_2_H_5_	CH_3_	170.6
10	CH_3_	C_2_H_5_	OCH_3_	132.2
11	H	C_2_H_5_	H	76.9
12	CH_3_	C_2_H_5_	H	66.1
13	C_2_H_5_	C_2_H_5_	H	60.3
14	NO_2_	C_2_H_5_	H	56.3
15	C(CH_3_)_3_	C_2_H_5_	H	50.1

**Fig. 7:**
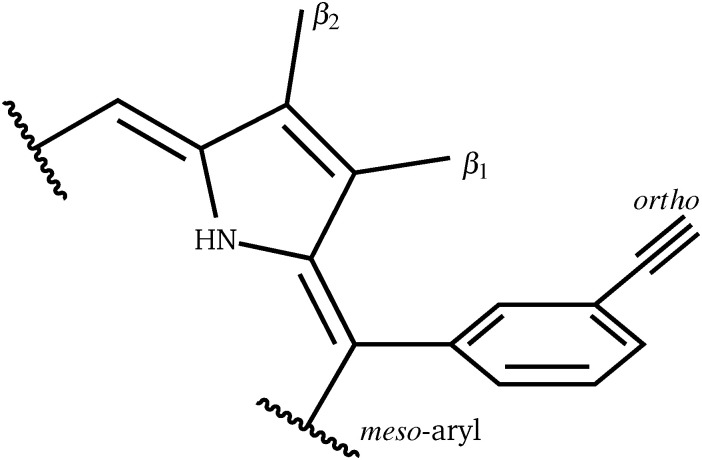
Definition of the β_1_, β_2_ and *meso* positions.

Perhaps counterintuitively, increasing the steric bulk at β_1_ (the closest pyrrolic group to the aryl, Fig. 7) results in a decrease in rotational barriers for the aryl substituents corresponding to P1. For β_1_ = H and CH_3_ the porphyrin remains planar, while bulkier groups induce a steric clash, causing buckling of the porphyrin ring, destabilizing the starting minimum. This prebuckling behaviour lifts the aryl ring out of plane in an opposing direction to that in which the β-substituents are forced, so rotation becomes easier, as all pathways require the same degree of buckling for rotation to occur. In summary, increased bulk at the β_1_ positions destabilizes the starting minimum by forcing a buckled conformation, reducing the barrier for rotation by up to 46.7 kJ mol^−1^. It is worth noting that the idea of destabilizing the starting minimum to improve the activity shares similarity with the steric nature of the bite angle in catalysis.^63,64^

**Fig. 8:**
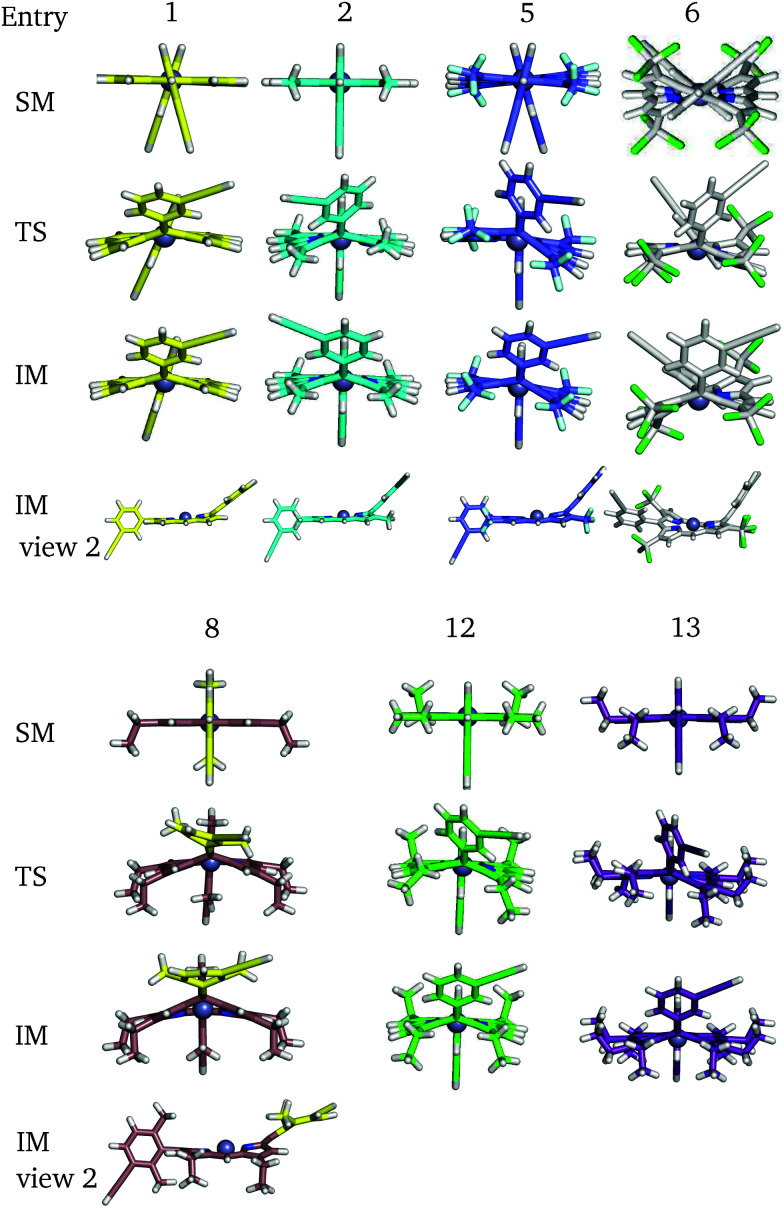
Some key stationary points in the flipping paths with different substituents, corresponding to Table 4.

Now considering β_2_ substitution, two different trends are observed in the β_2_-H and β_2_-C_2_H_5_ analogues. With low steric bulk in the β_1_ position and increasing bulk at the β_2_ position the same trend appears as above: pre-buckling in the starting minimum reduces the energy barrier for aryl rotation (Table 4: 1 + 11, 2 + 12, 2 + 14). With greater steric bulk at β_1_ and increasing bulk at the β_2_ position, the effect vanishes, and even reverses (Table 4: 4 + 14, 7 + 15), which might result from the repulsion between β_1_ and β_2_, inhibiting β_1_ from moving into the optimal position at the TS. In addition to porphyrin monomer variations, the β_2_-groups in P2 have all been substituted with β_2_-C_2_H_5_ to correlate how substituents affect the flipping of a porphyrin unit in the trimer (Table 5). It is observed that upon reducing the size and the polarity of the β_2_-group in P2, the intrinsic energy barrier remains almost unchanged. However, Δdiff and Δsolv are significantly reduced and the barrier comes down to 80.1 kJ mol^−1^, supporting the hypothesis we made in the last section. Hence, the β_2_-ester and β_2_-alkyl groups in P1 and P2 promote *meso*-aryl bond rotation in P1, but inhibit monomer flipping in P2.

**Table 5:** Potential energy of each stationary point relative to the starting minimum (SM) along the rotational pathway of P2 with β_2_-C_2_H_5_ as substituents (kJ mol^−1^)

	Δ*E*	Δdiff	Δsolv	ΔZPE	Final
SM	0.0	0.0	0.0	0.0	0.0
TS1^a^	78.2	+2.6	+0.1	−1.6	79.2
IM1	62.7	+2.9	0.0	−3.5	62.1
TS2	78.1	+3.0	+1.0	−2.0	80.1

a By replacing all the β_2_-substituents with β_2_-C_2_H_5_, the porphyrin trimer becomes symmetric, and TS1 and TS4, IM1 and IM3, TS2 and TS3, and SM and FM become identical within the pairs.

Substitution at the 2,6-aryl positions is a common strategy for preventing *meso*-substituent scrambling in the rational synthesis of asymmetric porphyrins, which would otherwise lead to lower yields and difficulties in chromatographic separation.^65–67^ It is generally accepted that the *ortho*-substitution of the aryl ring prevents coplanarity of the aryl ring and the pyrrole, which would stabilize the cationic azafulvenium by aromatization. Hence we investigated the rotational energy barrier for the 2,6-dimethyl substituted aryl porphyrin analogues, which was found to be remarkably high, namely 182.7 and 170.6 kJ mol^−1^ (Table 4 entries 8 + 9). These calculations agree with synthetic efforts, confirming that rotation at room temperature is extremely unlikely for the dimethyl analogue. For the *O*-methyl analogue (Table 4 entry 10) a lower barrier of 132.2 kJ mol^−1^ is predicted. This intermediate result fits with the observation that, in some cases, synthetic scrambling occurs despite the presence of 2,6-aryl substitution. In this case the electron donating nature of the *O*-methyl moieties may also play an important role in the stabilisation of the cationic azafulvenium, thereby facilitating scrambling.

## 4 Conclusions

We have synthesized a novel asymmetric porphyrin, ZnBAP_*m*_, and its cyclic trimer, Zn_3_TRI_*m*_. Calculations beginning with empirical force fields, and extending to explicit consideration of electronic structure, were used to predict the flipping pathway for the *meso*-aryl group in both the monomeric and trimeric species. The calculated energy barrier is 63.1 kJ mol^−1^, in good agreement with the results from variable temperature NMR spectroscopy, which yields a value of 65.4 kJ mol^−1^. The flipping pathway of a ZnBAP_*m*_ monomer in its trimer has also been predicted at the same levels of theory. The corresponding barrier is found to be much higher than for *meso*-aryl flipping in the monomer, in agreement with the NMR analysis. We carried out a set of calculations to systematically investigate how different substituents at the *meso* and β positions affect the dynamic behaviour. The results show that steric effects in certain locations can effectively facilitate the flipping. A methyl group at the *ortho*-position of *meso*-aryl is predicted to increase the barrier by up to 170 to 180 kJ mol^−1^, while a methoxyl group increases the barrier to 132.2 kJ mol^−1^. These results will be used to guide the design and synthesis of porphyrin oligomers in future experiments.

## Supplementary Material

CP-017-C5CP04636J-s001

CP-017-C5CP04636J-s002

CP-017-C5CP04636J-s003
