# Supplementary material for: How to make a porphyrin flip: dynamics of asymmetric porphyrin oligomers
Source: Phys Chem Chem Phys. 2015 Sep 15;17(40):27094–102. doi: 10.1039/c5cp04636j (PMC11745083; doi:10.1039/c5cp04636j)
Supplement: CP-017-C5CP04636J-s001 [file CP-017-C5CP04636J-s001.pdf]

Electronic Supplementary Material (ESI) for Physical Chemistry Chemical Physics.
This journal is © the Owner Societies 2015

I. SYNTHESIS

3-ethynyl benzaldehyde was prepared from 3-bromo benzaldehyde according to literature methods.[1]

A. 3-((trimethylsilyl)ethynyl)benzaldehyde

$PdCl_2(PPh_3)_2$ (1.0 g, 1.33×10^{-3} mol,), CuI (470 mg, 2.4×10^{-3} mol) and 3-bromobenzadehyde (14.3 ml, 20.8 g, 0.114 mol) in dry THF (100 ml) and dry Et_3N (15 ml) were subjected to three freeze-pump-thaw cycles before trimethylsilyl ethene was added *via* syringe under N_2. The reaction mixture was stirred at reflux for six hrs before cooling to room temperature. The product was extracted into DCM (100 ml) and washed with water (3×150 ml). The solvent was then removed under reduced pressure before FCC (silica, 5% EtOAc PET ether 40-60) afforded a yellow oil (20.7g, 0.103 mol, 91%).

^{1}H NMR (400 MHz, $CDCl_3$) δ= 10.02 (s, 1H), 7.99 (td, J = 1.6, 0.5 Hz, 1H), 7.85 (dt, J = 7.7, 0.5 Hz, 1H), 7.73 (m, J = 7.7, 1.6 Hz, 1H), 7.50 (t, J = 7.7 Hz, 1H), 0.30 (s, 9H).

B. 3-ethynyl benzaldehyde

3-((trimethylsilyl)ethynyl)benzaldehyde was dissolved in DCM (100 ml) before addition of K_2CO_3 (20 g) and MeOH (50 ml). After washing with water (3×100 ml) the solvent was dried and removed under reduced pressure to yield a yellow powder. Hot recrystallisation from hexane produced the product as off-white crystals (12.8 g, 0.0955 mol, 93%).

^{1}H NMR (400 MHz, $CDCl_3$): δ= 10.02 (s, 1H), 8.02 (s, 1H), 7.89 (d, J = 7.7 Hz, 1H), 7.76 (d, J = 7.7 Hz, 1H), 7.53 (t, J = 7.7 Hz, 1H), 3.19 (s, 1H).

C. Dimethyl 3,3′-(5,15-bis-(3-ethylphenyl)-8,12-dihexyl-3,7,13,17-tetramethyl zinc porphyrin-2,18-diyl) dipropionate

2,2′-(methylenebis(4-hexyl-3-methyl-1H-pyrrole-5,2-diyl))bis(1-phenylethane-1,2-dione) (kindly prepared by Mr C Sporikou according to literature methods[2,3]) (2.00 g, 3.30 mmol), dimethyl 3,3′-(methylenebis(4-methyl-5-(2-oxo-2-phenylacetyl)-1H-pyrrole-2,3-diyl))dipropionate(Kindly prepared by Mr C Sporikou) (2.01 g, 3.30 mmol) and triethylamine (3 ml) in dry THF (50 ml) were stirred under $H_{2(g)}$ for 2 hrs. The reaction mixture was then filtered through celite and the solvent removed to yield an off-white foam. After TFA (10 ml) had been exposed to $N_{2(g)}$ degassing it was added by cannula to the reaction vessel and stirred for 20 mins.

To the resultant deep red solution, a $N_{2(g)}$ degassed 3-ethynyl benzaldehyde (0.99 g, 7.60 mmol) in dry MeOH (40 ml) solution was added *via* cannula at -20 °C and stirred at this temperature for 2 hrs. DDQ (2.00 g) was then added and stirred under $N_{2(g)}$ for 1 hr. The reaction mixture was passed through a plug (Al_2O_3, DCM) before FCC (SiO_2, DCM → DCM:EtOAc (5%)). Product was collected as the second of three porphyric bands, dried and solvent removed prior to layered addition recrystallisation (MeOH on $CHCl_3$) yielded the product as purple crystals (379 mg, 0.42 mmol, 11%)

^{1}H NMR (500 MHz, $CDCl_3$): δ= 10.29 (s, 1H), 10.272 (s, 1H), 8.24 (s, 2H), 8.08 (d, J=7.6 Hz, 2H), 7.97 (d, J=7.6 Hz, 2H), 7.74 (t, J=7.6 Hz, 2H), 4.39 (t, J=8.0 Hz, 4H), 3.99 (t, J=8.0 Hz, 4H), 3.69 (s, 6H), 3.22-3.15 (m, 6H), 2.55 (s, 6H), 2.53 (s, 6H), 2.19 (quin, J=7.6 Hz, 4H), 1.757 (quin, J=7.4 Hz, 4H), 1.50 (quin, J=7.6 Hz, 4H), 1.38 (quin, J=7.4 Hz, 4H), 0.92 (t, J=7.6 Hz, 6H), -2.42 (s, 2H).

MALDI-ToF expected: 907.21 m/z; observed: 907.20 m/z.

D. P1, Dimethyl 3,3′-(5,15-bis(3-ethynylphenyl) -8,12-dihexyl-3,7,13,17-tetramethyl zinc porphyrin-2,18-diyl) dipropionate

Porphyrin (254 mg) and zinc acetate (300 mg) in DCM (20 ml) and MeOH (0.2 ml) were stirred 2 hrs, filtered and solvent removed. Layered addition recrystallisation (MeOH on $CHCl_3$) yielded purple/pink crystals (251 mg, 92 %).

^{1}H NMR (500 MHz, $CDCl_3$): δ= 10.24 (s, 1H), 10.22 (s, 1H), 8.26 (s, 2H), 8.10 (d, J=7.6 Hz, 2H), 7.97 (d, J=7.6 Hz, 2H), 7.74 (t, J=7.6 Hz, 2H), 4.37 (t, J=8.0 Hz, 4H), 3.98 (t, J=8.0 Hz, 4H), 3.72 (s, 6H), 3.22-3.15 (m, 6H), 2.52 (s, 6H), 2.50 (s, 6H), 2.20 (quin, J=7.6 Hz, 4H), 1.77 (quin, J=7.4 Hz, 4H), 1.52 (quin, J=7.6 Hz, 4H), 1.42 (quin, J=7.4 Hz, 4H), 0.94 (t, J=7.6 Hz, 6H).

^{13}C NMR (126 MHz, Toluene): δ= 172.51, 147.52, 147.25, 146.63, 145.68, 144.29, 143.41, 141.45, 138.09, 137.56, 136.64, 133.41, 131.61, 118.01, 97.58, 97.42, 77.67, 50.65, 36.77, 33.45, 32.02, 30.10, 26.65, 22.82, 21.74, 15.42, 15.22, 13.94.

MALDI-ToF expected: 970.58 m/z; observed: 970.10 m/z.

E. P2

A 250 ml rbf charged with dry DCM (100 ml), P1 (58 mg, 0.06 mmol) and TPyT (50 mg, 0.18 mmol) was stirred under dry air for 5 mins before addition of CuCl (0.40 g, 3.0 mmol) and TMEDA (0.45 ml, 0.35 g, 3.0 mmol) and subsequent room temperature stir under dry air. The reaction mixture was concentrated, washed with water (3 $\times$ 500 ml) before being dried over $MgSO_4$, solvent removed and chromatographed through silica (Hexanes/EtOAc 90:10). The fraction containing P2 was further purified by FFC through alumina (DCM). The product was obtained as a purple residue (8.3 mg, 2.9 μmol, 15%)

^{1}H NMR (500 MHz, Tol, 363K): δ= 10.13 (s, 3H), 10.05 (s, 3H), 9.09 (s, 6H), 7.72 (d, J=7.9 Hz, 6H), 7.41 (d, J=7.6 Hz, 6H), 7.24 (t, J=7.6 Hz, 6H), 4.22-4.20 (m, 12H), 3.84-3.82 (m, 12H), 3.22 (s, 18H), 3.02-2.90 (m, 12H), 2.57 (s, 12H), 2.53 (s, 12H), 1.61-1.59 (m, 12H), 1.19 -1.17 (m, 12H), 0.87-0.81 (m), 0.73-0.70 (m, 16H).

MALDI-ToF expected: 2905.68 m/z; observed: 2905.77 m/z.

^{13}C NMR (126 MHz, Tol) δ= 173.22, 148.57, 148.31, 147.61, 146.70, 145.73, 144.89, 144.70, 142.87, 140.97, 139.87, 134.83, 130.73, 121.81, 118.27, 98.26, 98.17, 84.15, 76.41, 51.36, 37.66, 34.36, 32.75, 30.98, 30.75, 27.71, 23.54, 22.77, 16.43, 14.67, 1.80.

F. P3, ZnBAP$_b$, 5,15-bis(3-ethynylphenyl)-2,8,12,18-tetrahexyl-3,7,13,17-tetramethyl zinc porphyrin

The free base porphyrin was collected from the statistical synthesis of P1, recrystalised (MeOH on $CHCl_3$) and obtained as purple crystals (241 mg, 0.27 mmol, 7%) and treated with zinc acetate as above to yield purple crystals (237 mg, 92%).

^{1}H NMR (500 MHz, Tol, 363K): δ= 10.38 (s, 2H), 8.35 (s, 4H), 7.94 (d, J=7.8 Hz, 4H), 7.86 (d, J=7.9 Hz, 4H), 7.43 (t, J=7.9 Hz, 4H), 4.07 (t, J=7.7 Hz, 8H), 2.88 (s, 2H), 2.63 (s, 12H), 2.36 (quin, J=7.8 Hz, 8H), 1.87 (quin, J=7.8 Hz, 8H), 1.59 (quin, J=7.8 Hz, 8H), 1.46 (quin, J=7.8 Hz, 8H), 1.00 (t, J=7.8 Hz, 12H).

MALDI-ToF expected: 966.72 m/z; observed: 966.24 m/z.

II. COSY AND TOCSY OF P1

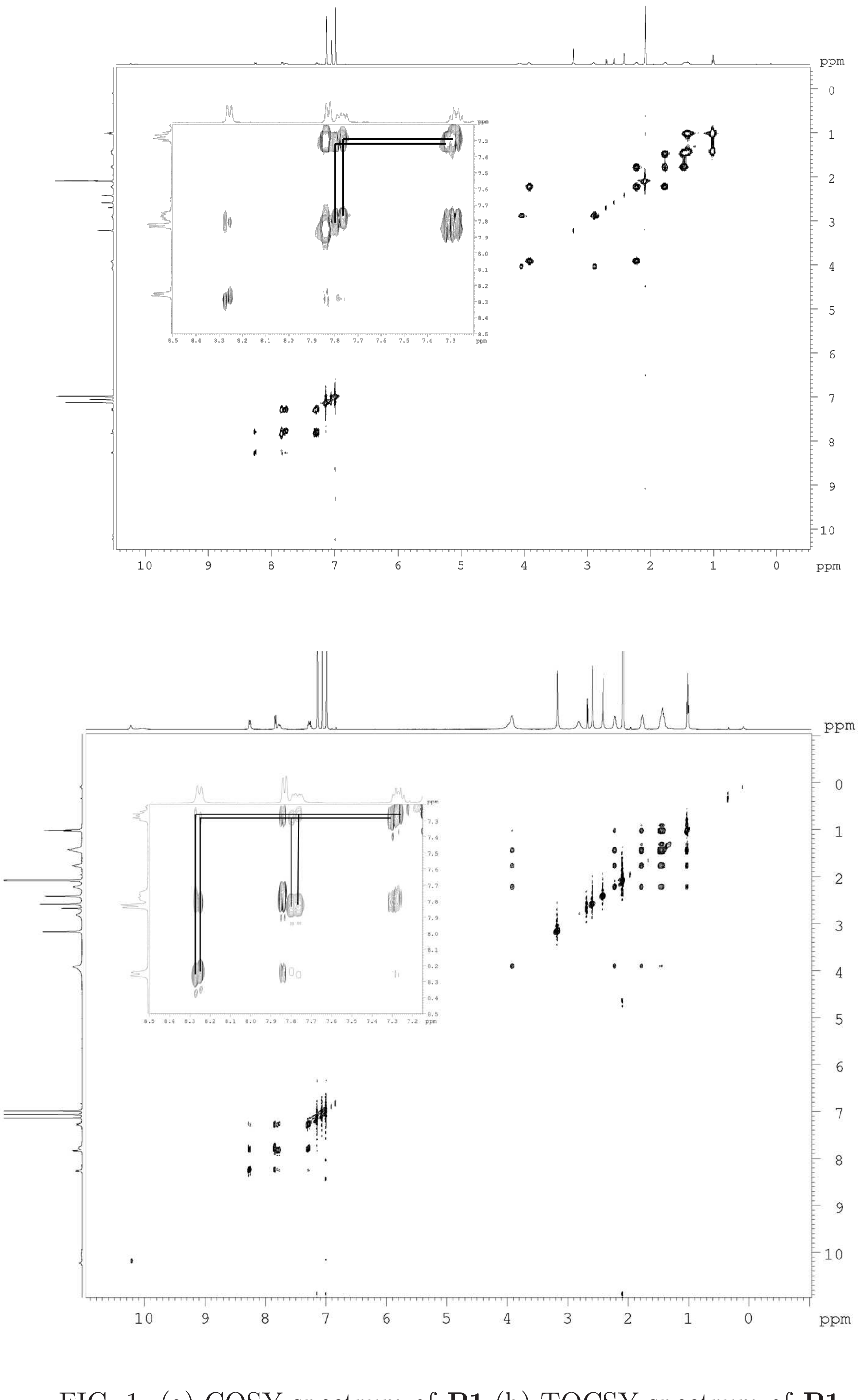

FIG. 1. (a) COSY spectrum of **P1** (b) TOCSY spectrum of **P1**

III. VT-NMR SPECTRUM OF P2 WITH TPYT

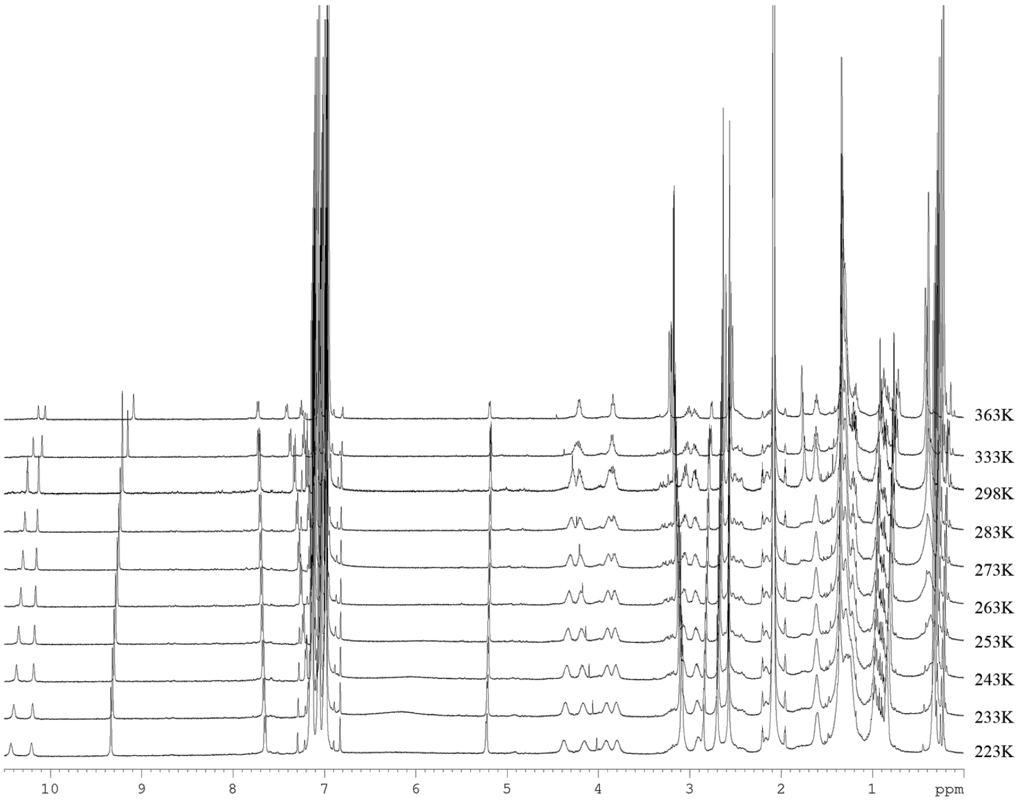

FIG. 2. VT-NMR spectrum of **P2** with TPyT

IV. NMR SPECTRUM OF P1 AT 298 K IN D$_8$-TOLUENE

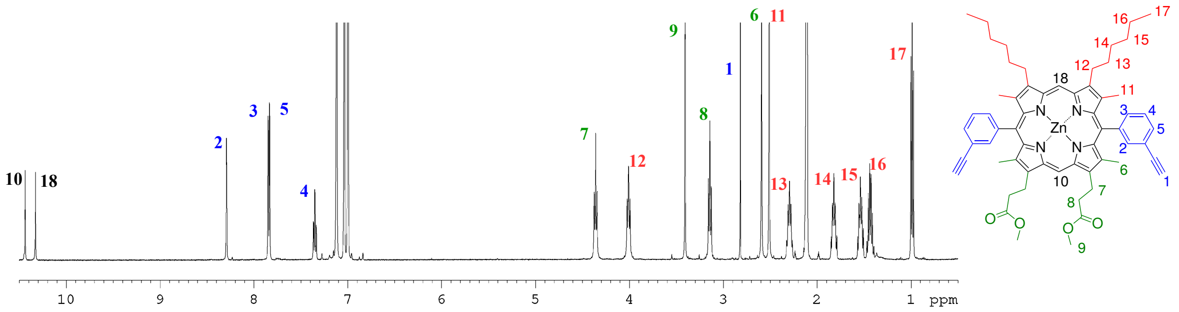

FIG. 3. NMR spectrum of **P1** at 298 K in d$_8$-toluene

V. IMAGINARY VIBRATIONAL FREQUENCIES OF TRANSITION STATES FOR P1 AND P2

P1: TS1: 79.9 cm^{-1}, TS2: 68.9 cm^{-1}. **P2**: TS1: 67.3 cm^{-1}, TS2: 83.3 cm^{-1}, TS3: 78.6 cm^{-1}, TS4: 75.3 cm^{-1}.

VI. HOMO ORBITALS OF P2 IN SM, IM1, IM2 AND IM3

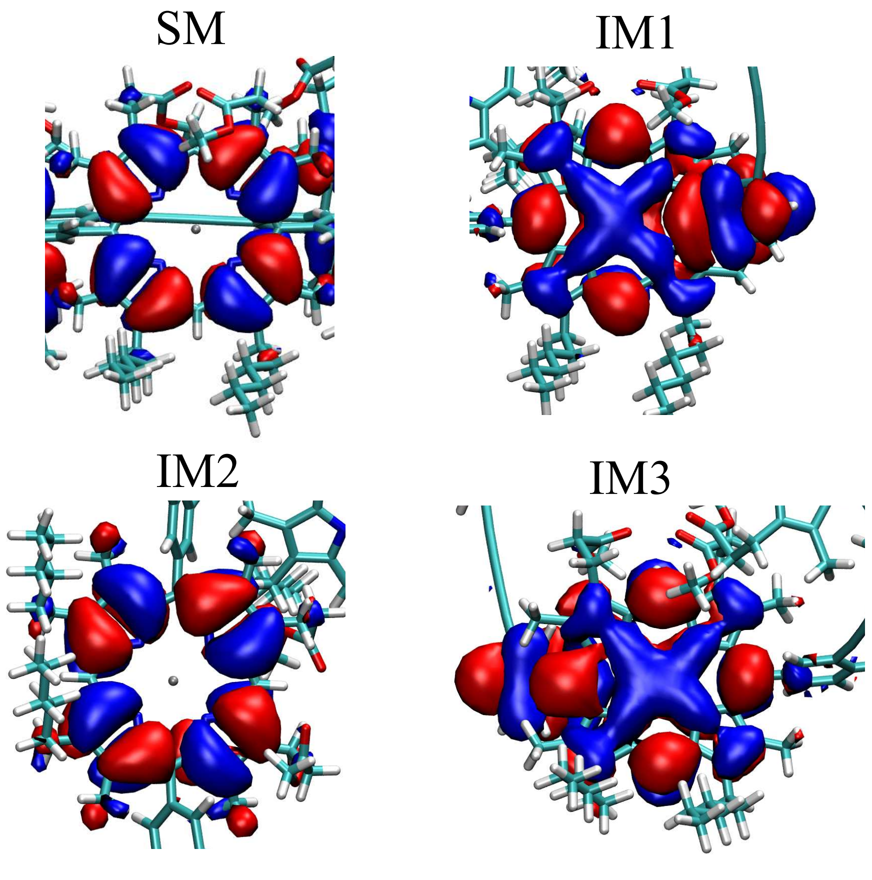

FIG. 4. The homo orbitals of **P2** in SM, IM1, IM2 and IM3. For clarity, only the orbitals of flipping M1 is shown here.

VII. VIDEOS FOR THE P2 FLIPPING PATH

The online videos "view2.avi" and "view3.avi" correspond to the flipping path of **P2** that calculated using AMBER force field as an aid of visualization of fig. 5 in the main text. The starting points of videos correspond to SM (View 2) and SM (View 3) respectively. The parameters used are the same as described in the calculation details of the main text.

REFERENCES

[1] Ghosh, B.; Amado-Sierra, M. D. R. I.; Holmes, D.; Maleczka, R. E. *Org. Lett.* **2014**, *16*, 2318–2321.

[2] Helms, A.; Heiler, D.; McLendon, G. *J. Am. Chem. Soc.* **1992**, *114*, 6227–6238.

[3] Abdalmuhdi, I.; Chang, C. K. *J. Org. Chem.* **1985**, *50*, 411–413.
